# pH-Responsive Peptide Nanoparticles Deliver Macromolecules to Cells via Endosomal Membrane Nanoporation

**DOI:** 10.1021/acsnano.4c07525

**Published:** 2024-12-09

**Authors:** Eric Wu, Ains Ellis, Keynon Bell, Daniel L. Moss, Samuel J. Landry, Kalina Hristova, William C. Wimley

**Affiliations:** † Department of Biochemistry and Molecular Biology, Tulane University School of Medicine, New Orleans, Louisiana 70112, United States; ‡ Chemistry-Biology Interface Program, Johns Hopkins University, Baltimore, Maryland 21218, United States; § Institute for NanoBioTechnology, Johns Hopkins University, Baltimore, Maryland 21218, United States; ∥ Department of Materials Science and Engineering, Whiting School of Engineering, Johns Hopkins University, Baltimore, Maryland 21218, United States

**Keywords:** peptide, nanoparticle, nanopore, protein delivery, drug delivery

## Abstract

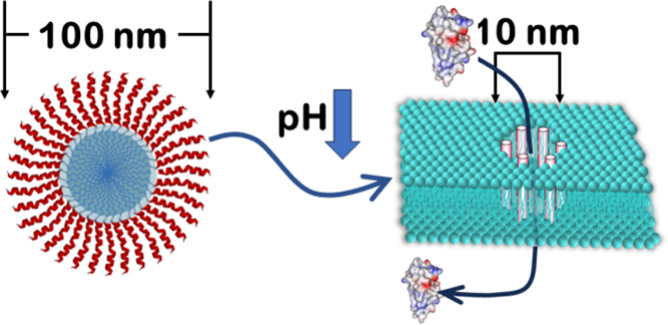

The synthetically evolved pHD family of peptides is known to self-assemble into macromolecule-sized nanopores of 2–10 nm diameter in synthetic lipid bilayers, but only when the pH is below ∼6. Here, we show that a representative family member, pHD108, has the same pH-responsive nanopore-forming activity in the endosomal membranes of living human cells, which is triggered by endosomal acidification. This enables the cytosolic delivery of endocytosed proteins and other macromolecules. Acylation of either peptide terminus significantly decreases the concentration of peptide required for macromolecule delivery to the cell cytosol while not causing any measurable cytotoxicity. Longer acyl chains are more effective. The N-terminal palmitoylated C16-pHD108 is the most potent of all of the acyl-pHD108 variants and readily delivers a cytotoxic enzyme, fluorescent proteins, and a dye-labeled dextran to the cell cytosol. C16-pHD108 forms stable monodisperse micellar nanoparticles in a buffer at pH 7 with an average diameter of around 120 nm. These nanoparticles are not cytolytic or cytotoxic because the acylated pHD peptide does not partition from the nanoparticles into cell membranes at pH 7. At pH 5, the nanoparticles are unstable, driving acylated pHD108 to bind strongly to membranes. We hypothesize that passive endocytosis of macromolecular cargo and stable peptide nanoparticles, followed by endosomal acidification-dependent destabilization of the nanoparticles, triggers the nanopore-forming activity of acylated pHD peptides in the endosomal membrane, enabling internalized macromolecules to be delivered to the cytosol.

## Introduction

Many of the top therapy targets identified by the National Cancer Institiute^1,2^ are intracellular proteins involved in protein–protein interaction (PPI) networks that function pathologically in cancer. The search for membrane-permeant small molecule drugs to target PPI interfaces has yielded few successes, likely because of the large, distributed surface areas of these interfaces.^3^ As a result, many signaling mediators—which include transcription factors such as c-Myc, NF-kB, Ras family proteins, and many others—are considered to be nearly“undruggable”.^4^ Peptides and proteins, including interface-targeting peptides as well as antibodies and nanobodies, can readily modulate PPIs with very high specificity^5−10^ but they have the disadvantage of being very difficult to deliver to the cell cytosol by any means other than through gene or mRNA transfection. Thus, the difficulty of cytosolic delivery of peptides and proteins is a key impediment to the development of potential intracellular peptide and protein therapeutics for cancer and many other diseases.^4^

Cytosolic delivery of macromolecules requires their passage across a membrane: either direct passage across the plasma membrane or uptake into a membrane-bound endocytic compartment followed by passage across the compartment membrane. Currently there exist a number of methods for macromolecule delivery. Physical disruption, such as electroporation, can enable protein delivery in some cell types.^11^ A few families of cell-penetrating peptides deliver cargos to cells through predominantly direct plasma membrane translocation.^12−15^ However, most delivery mechanisms require endosomal uptake followed by passage across the endosomal membrane. This can be driven by viral fusion proteins or bacterial toxins or by other membrane-disrupting reagents, such as peptides, proteins, nanoparticles, or polymers.^16^ Some cationic cell-penetrating peptides cause endosomal release,^14,17,18^ mostly by acting nonspecifically on endosomal membranes via interfacial activity,^19^ requiring high local concentrations. Even commercial protein transfection or “profection” products, mostly sold for antibody delivery, have limited capabilities, sometimes displaying a complete inability to deliver functional proteins into cells.^20^

We previously reasoned that the release of endocytosed macromolecules into the cytosol could be accomplished by peptides if they could be evolved to be triggered to self-assemble into macromolecule-sized nanopores in endosomal membranes upon the expected acidification. Toward this goal, we used synthetic molecular evolution^13,21−24^ to evolve pH-responsive peptides that potently self-assemble into macromolecule-sized nanopores (i.e., pores ≥1 nm diameter) in synthetic bilayers in response to a decrease in pH that mimics the acidification that occurs in early endosomes. The evolution of this peptide lineage proceeded through several generations. Starting with a library that was based on the sequence of the generic membrane permeabilizing bee venom peptide melittin,^25^ we evolved MelP5, a potent equilibrium pore-forming peptide that forms macromolecule-sized pores in synthetic bilayers at all pH values, but only at high peptide concentration.^26,27^ In the subsequent generation, we evolved peptides from a MelP5-based library that form nanopores with high potency, but only at acidic pH. We named this family the “pHD peptides”, which, even at very low concentrations, cause pH-triggered nanopore formation in lipid vesicles made from fluid-phase phosphatidylcholine lipids.^28−30^ Specifically, in lipid bilayers at pH ≤ 5.5, the pHD peptides self-assemble into 2–10 nm diameter and larger nanopores that enable the passage of macromolecules across the membrane.^28−30^ In this work, we show that acylated pHD peptides self-assemble into stable nanoparticles in a neutral solution but have the same pH-responsive behavior in the endosomes of human cells, forming nanopores delivering endocytosed macromolecules to the cell cytosol at acidic pH.

## Results

### pHD Peptides Self-Assemble into Nanopores

**Figure 1:**
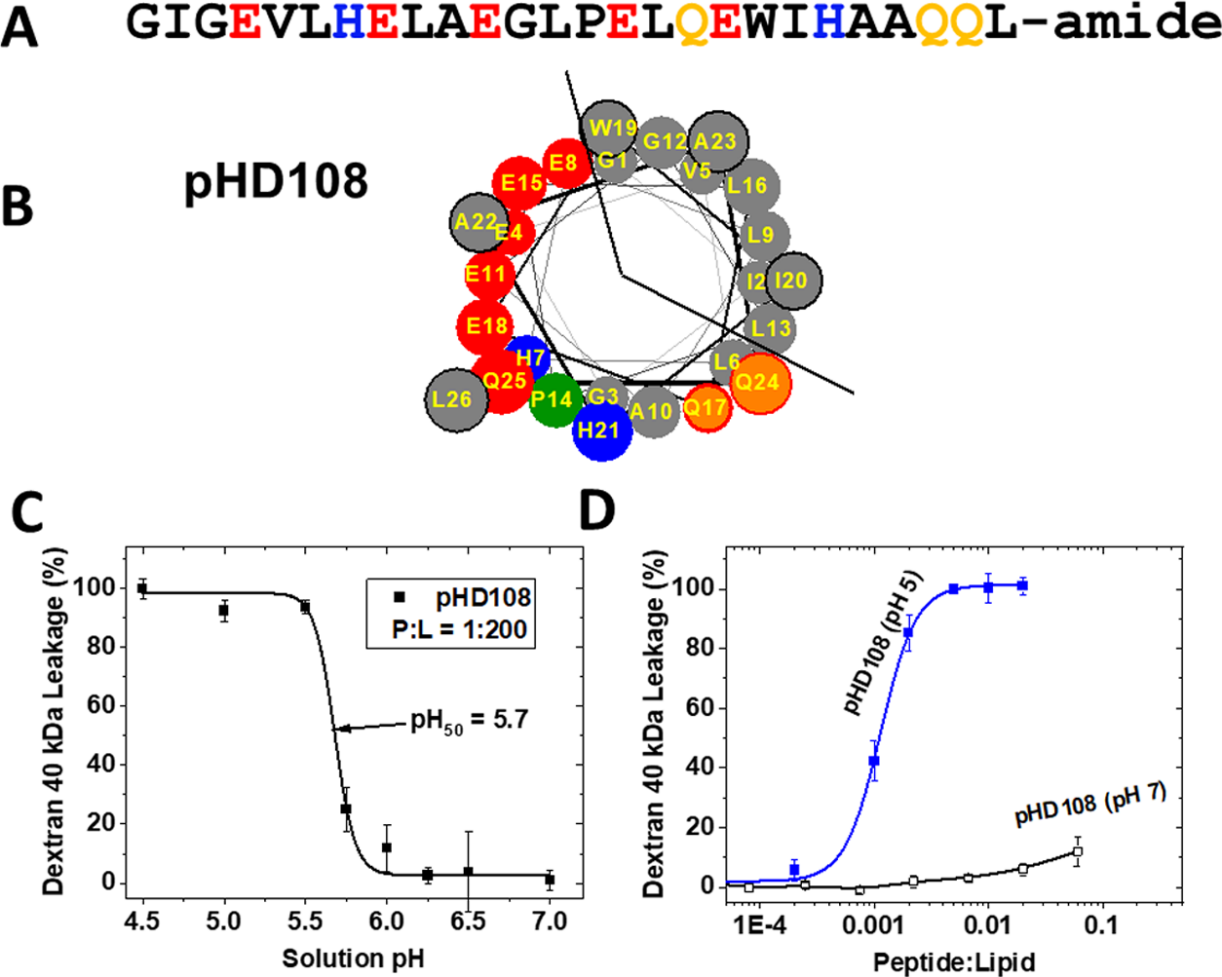
Synthetically evolved nanopore-forming peptide pHD108. (A) The amino acid sequence of pHD108 is shown. In this work, we use both l- and d-amino acid versions of pHD108. (B) Helical wheel diagram for pHD108, showing nonpolar residues in gray, basic residues in blue, acidic residues in red, and polar residues in orange. The P14 residue, which is essential for activity, is shown in green. (C) Nanoporation activity of pHD108 in synthetic lipid vesicles made from 1-palmitoyl-2-oleoly phosphatidylcholine (POPC), versus pH.^28,30^ Nanoporation is measured by the release of a 40 kDa dextran from liposomes. (D) Concentration dependence of nanoporation activity^28,30^ in synthetic liposomes.

The amino acid sequence and helical wheel diagram for the nanopore-forming peptide, pHD108, are shown in Figure 1. All of the pHD peptides that we tested have similar nanoporation activity in synthetic liposomes made from fluid-phase phosphatidylcholine lipids,^28−30^ but in this work, we focus our studies on pHD108, the best-studied member of the family, Figure 1A. This nanopore-forming peptide forms amphipathic α-helices with distinct faces, one that is very hydrophobic and one that is highly polar and charged, as shown in Figure 1B. Like most of the pHD peptides, pHD108 has five acidic amino acids, two basic amino acids, and a few additional polar amino acids. These charged and polar residues are aligned broadly along one surface of the amphipathic α-helix, as shown in the helical wheel diagrams in Figure 1B.^23,28−32^ We have previously described the properties of pHD108 in fluid-phase synthetic lipid bilayers made from 1-palmitoyl-2-oleoyl-phosophatidylcholine (POPC).^28−30^ At pH < 5.5, pHD108 binds to bilayers, folds into the α-helical secondary structure, and self-assembles into membrane-spanning nanopores that release a 40 kDa dextran macromolecule from the vesicles.^28−30^ At a system peptide to lipid molar ratio (P/L) of 1:200, the pHD peptides have a pH-midpoint (pH_50_), or effective p*K*_a_, of around pH 5.7, Figure 1C. At pH 7, the nanoporation activity of pHD108 is minimal, even at high P/L, Figure 1D. At pH 5, the concentration midpoint for nanoporation is around P/L = 1:1000, which is extremely potent for this kind of activity.^33^ Nanoporation activity requires only about 70 bound peptides per vesicle.^30^ We have recently shown, for a family of very closely related peptides called macrolittins, that the multiple polar and charged groups on the polar surface of the helix, Figure 1B, stabilize the membrane-spanning nanopore by forming a cooperative network of water- and lipid-bridged hydrogen bonds across the bilayer.^34^

### Development of a High-Throughput Protein Delivery Assay

**Figure 2:**
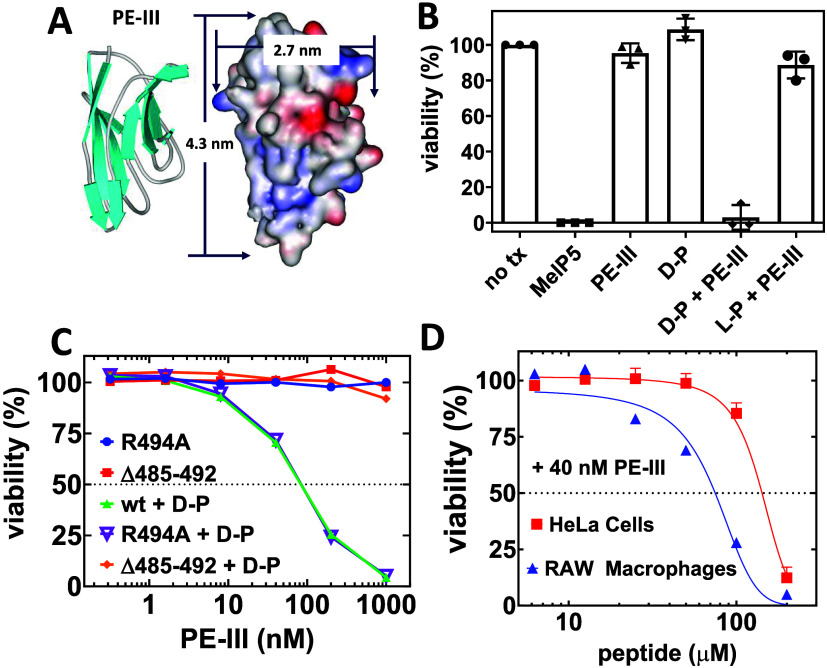
Development of an assay to measure the delivery of the PE-III protein to live cells. (A) The PE-III protein cargo is ∼25 kDa and has major and minor axes of 4.3 and 2.7 nm, respectively. (B) Fate of HeLa cells incubated with peptides, PE-III protein, or a combination. MelP5 was used at 25 μM, pHD108 was used at 200 μM, and PE-III was used at 40 nM. Cell viability was measured 48 h after incubation to allow for apoptosis to occur. Notation: l-P and d-P are l- and d-amino acid versions of pHD108. (C) Delivery of PE-III mutants is measured by Alamar Blue detection of cell viability. The R494A mutant, which is biochemically active but less stable, was tested with and without pHD108. The Δ485–492 mutant, which is biochemically inactive, was also tested with and without pHD108. (D) The PE-III delivery assay was tested on HeLa cells and RAW macrophages using 40 mM PE-III and a range of pHD108 concentrations.

To study the macromolecule delivery activity of pHD108, we developed a sensitive, high-throughput-capable assay for the peptide-mediated delivery of a protein to the cytosol of living cells. For this, we use PE-III, the third domain of the *Pseudomonas* exotoxin. This 25 kDa enzyme ADP ribosylates ribosomal elongation factor 2, thereby blocking protein synthesis and triggering apoptosis. Lacking domains I and II, which are responsible for uptake and delivery of the exotoxin,^35^ the PE-III enzyme does not enter cells spontaneously, as we verify below. Thus, apoptosis will occur only if an exogenous agent, such as a peptide, enables cytosolic delivery of the 4 × 3 nm PE-III protein, Figure 2A. Because pHD108 alone has no effect on cell viability (see below), we can measure viability after 48 h as a reporter of apoptosis, which results from the successful cytosolic delivery of PE-III.

The PE-III assay has the benefit of producing a clear, quantifiable outcome: the midpoint of the cell death versus peptide concentration curve, which we refer to as EC_50_. Comparison of peptide concentration midpoints requires a constant concentration of PE-III in each experiment. EC_50_ values are used below to compare variants of pHD108. Used in a screen, the PE-III assay can produce a clear binary yes-no output, although we do not use the assay for screening in this work. The major disadvantage of the PE-III assay to detect delivery is that only a few molecules of active PE-III delivered to the cytosol^36^ will produce a positive result. To partly mitigate this effect in the assays described below, we use a constant 40 nM PE-III. At such a low concentration, each endosome will contain an average of fewer than one PE-III molecule if we assume passive entrapment of the cargo (see the Supporting Information).

HeLa human cervical cancer cells were incubated with or without 40 nM PE-III and/or 200 μM d-amino acid pHD108 (d-pHD108) at 37 °C for 48 h prior to determining the cell viability by measuring the activity of energized mitochondria using Alamar Blue, Figure 2B. The no treatment (no tx) control group contained cells but no peptide or PE-III enzyme. The Alamar Blue intensity of this group was defined as 100% viability. Media alone without cells was defined as 0% viability. To show that the assay was properly reporting on cell death, we used 25 μM of the potent membrane lytic peptide MelP5,^26^ which reduced the Alamar Blue signal of cells to the media-only background. Next, we incubated cells with either 40 nM PE-III alone or with 200 μM d-amino acid pHD108 alone and observed no effect on cell viability, Figure 2B. When 200 μM d-pHD108 and 40 nM PE-III were added together, essentially 100% loss of cell viability was observed, showing that PE-III was effectively delivered to the cytosol by the pHD peptide. Complete loss of Alamar Blue activity demonstrates that PE-III delivery occurred to essentially every cell in the well, with a limit of detection of about 1–2% of the viable cells. Finally, when we incubated cells with 40 nM PE-III and 200 μM l-pHD108 made from protease-susceptible l-amino acids, we observed very little loss of cell viability. This observation provides strong support for the hypothesis that PE-III delivery occurs via endocytosis and that it is dependent on endosomal acidification because the l-amino acid peptide, but not the d-amino acid peptide, is susceptible to acidification-dependent proteolytic degradation which occurs in endosomal compartments.

To show the robustness of the assay, we tested it with two PE-III mutants: The R494A mutant, which has been shown to be less stable and more susceptible to proteolysis but enzymatically active,^37^ and the Δ485–492 mutant, which has a deleted surface loop^37^ and reduced or eliminated enzymatic activity because the deletion includes residues near the active site.^38^ We found that both wild type and R494A PE-III had nearly identical apoptosis activities in the pHD108-dependent delivery assay and that both depend completely on the presence of d-pHD108 for activity (Figure 2C). The enzymatically inactive Δ485–492 had no effect on cell viability even in the presence of 200 μM d-pHD108, showing that our cell viability assay reports directly on cytosolic enzyme activity resulting from the delivery of intact functional protein to the cytosol.

Finally, we tested PE-III delivery using human RAW macrophages to compare to HeLa cells, as shown in Figure 2D. d-pHD108-dependent PE-III delivery readily occurs in these macrophages with an EC_50_ for d-pHD108 of 75 μM compared to 120 μM in HeLa cells, showing that the activity of pHD108 is similar across different cell types. The lack of any residual Alamar Blue activity at high peptide concentrations in the presence of PE-III indicates that every RAW cell is subject to PE-III-induced apoptosis, a result that we also observed for HeLa cells.

### Acylation Drives a Dramatic Increase in Macromolecule Delivery Activity

**Figure 3:**
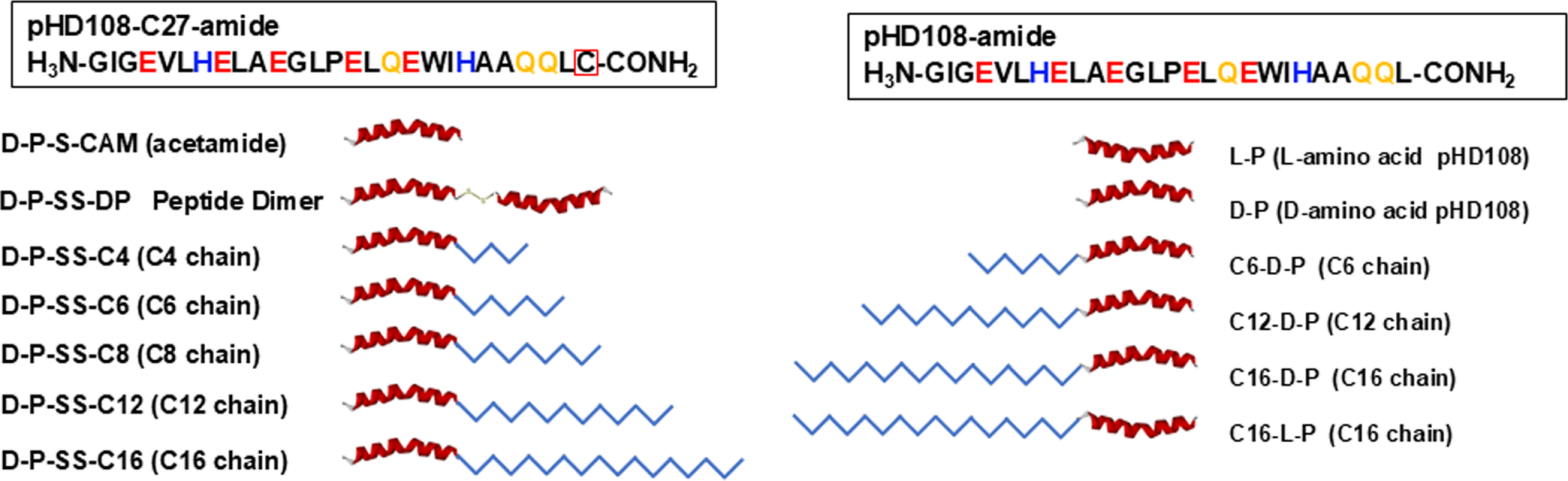
Variants of pHD108 were tested in this work. Two groups of acylated peptides were synthesized. (Left) pHD108 with a C-terminal cysteine had alkyl chains attached by a disulfide cross-link. This group includes a peptide with the sulfhydryl group alkylated with acetamide and a disulfide cross-linked peptide dimer. (Right) pHD108 with acyl chains attached to the amino-terminal amine by an amide bond. This group includes unmodified l- and d-pHD108, as well as l- and d-pHD108 with C16 chains. Notation: d-P, l-P: d- and l-amino acid pHD108. CN: Linear, saturated alkyl chain with N carbons. CN- at the start of the name indicates an N-terminal acylated peptide. −CN at the end of the name indicates a C-terminal modified peptide. These images are not drawn to scale—the acyl chain sizes are exaggerated for increased visibility.

The pHD peptides were evolved for pH-triggered nanopore formation at acidic pH but not for cell binding or endosomal uptake at pH 7. In the experiments above, the pHD peptides are likely to be passively taken up, explaining why a 200 μM peptide is required for activity. In the next stage of optimization, we added acyl groups to pHD108 with the intention of improving cell binding and uptake. We thus synthesized multiple acylated variants of pHD108 and measured their ability to deliver the PE-III protein to the cytosol of human cells compared to unmodified pHD108. For this part of the work, we tested two conjugation approaches: (i) linking of saturated thioacyl chains to a C-terminal cysteine by a disulfide bond which can be reduced by endosomal enzymes or (ii) linking of saturated fatty acids to the N-terminal amino group by an amide bond that is not subject to endosomal hydrolysis. We utilized saturated chains between 4 and 16 carbons on the C-terminus and saturated chains between 6 and 16 carbons on the N-terminus. Most conjugates were made with d-amino acid pHD108, and a few were made with l-amino acid pHD108 for comparison. We also made a disulfide cross-linked dimer of d-pHD108 that was not acylated. All of the acylated variants and control peptides that we synthesized and tested are shown in Figure 3.

**Figure 4:**
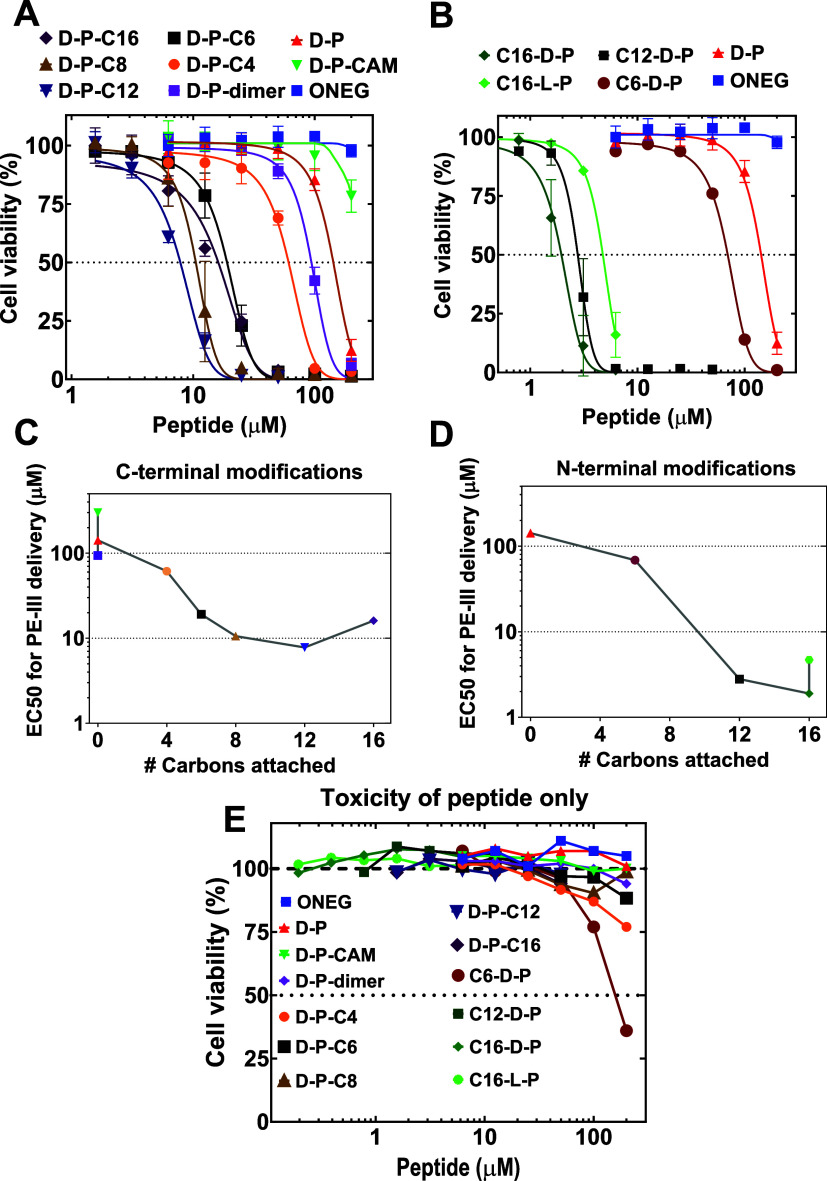
PE-III protein delivery by acylated pHD108 variants. (A, B) Delivery of PE-III by C-terminal variants (A) and N-terminal variants (B). HeLa cells were incubated with 40 nM PE-III and serially diluted pHD108 variants. Cytosolic PE-III causes apoptosis. To assay for delivery, cell viability was measured 48 h after treatment using Alamar Blue. The signal from cells with no treatment was defined as 100% viability. Media without cells was defined as 0% viability. Peptide variant notation is defined in Figure 3. ONEG is an unrelated peptide^39^ used as a control. (C, D) Concentration midpoint (EC_50_) values for the C-terminal variants (C) and N-terminal variants (D) determined from the data in panels (A, B). (E) Direct cytotoxicity of the pHD peptide variants in the absence of the PE-III cargo measured by Alamar Blue, 48 h after treatment with peptides as above.

All acylated variants of pHD108, Figure 3, were soluble in buffer at pH 7. To test for PE-III delivery by acylated pHD108 peptides, we incubated serially diluted peptides with HeLa cells in the presence of 40 nM PE-III in media at physiological pH. The concentration range used for each peptide was adjusted according to preliminary measurements of activity in this assay. We assessed PE-III delivery by measuring cell viability using Alamar Blue. Unmodified pHD108 had EC_50_ = 140 μM peptide in the presence of 40 nM PE-III. An unrelated negative control peptide called ONEG (PLGRPQLRRGQF-amide), which was previously shown to have no cellular activity,^39,40^ induced no measurable PE-III delivery/apoptosis over the same concentration range. The disulfide cross-linked dimer of pHD108 had EC_50_ = 93 μM, showing slightly more activity than the monomer. In all cases, acylation dramatically improved the delivery of PE-III, as quantified by the EC_50_, or the peptide concentration midpoint for PE-III-dependent apoptosis, Figure 4. PE-III delivery experiments enabled by variants with C-terminal acylation are shown in Figure 4A, and PE-III delivery enabled by variants with N-terminal acylation are shown in Figure 4B. Acylation at either terminus sharply shifted the concentration dependence of PE-III delivery to lower peptide concentrations. EC_50_ values for C-terminal variants are shown in Figure 4C, and EC_50_ values for N-terminal variants are shown in Figure 4D. The effect of acylation was significant for either terminus and for any chain length; however, longer chains generally provided greater activity and lower EC_50_ values. The most active C-terminal variant, d-pHD108-C12, had an EC_50_ of 8 μM, compared to 140 μM for the unmodified peptide. The N-terminally acylated pHD108 conjugates were somewhat more active than the equivalent C-terminal conjugates. The most active variant, overall, C16-d-pHD108, had an EC_50_ of 1.9 μM, which is a 74-fold increase in activity over unmodified pHD108.

We tested for direct toxicity of the acylated pHD peptides by incubating the same concentrations of acylated pHD peptide variants with cells in the absence of PE-III. Direct cytotoxicity of the acylated pHD peptides was essentially zero at concentrations at which the PE-III assay showed a complete loss of cell viability, Figure 4E. Thus, all of the apoptosis observed in the PE-III delivery assays was due to the cytosolic delivery of intact PE-III and not to the direct action of the acylated pHD peptide.

### C16-d-pHD108 Delivers Other Macromolecules to the Cytosol

Because the PE-III enzyme amplifies its effects in the cytosol, it does not report directly on the amount of cargo delivered to the cytosol. To demonstrate C16-d-pHD108-enabled delivery of a macromolecule to cells that does not amplify its signal, we next used confocal microscopy to measure the delivery of a dye-labeled dextran of 10 kDa to the cell cytosol. This dextran is a hydrated prolate ellipsoid with a hydrodynamic radius of ∼1.9 nm (diameter 3.8 nm) and an axial ratio of 3–4,^41,42^ dimensions that are similar to the PE-III protein, which has a hydrodynamic radius of about 1.7 nm (diameter 3.4 nm) and an axial ratio of about 1.6. We note that the equivalent hydrodynamic radius of PE-III and a 10 kDa dextran are verified by size exclusion chromatography, which showed that dextrans behave hydrodynamically like proteins that are about 4-fold larger in molecular weight.^43^

**Figure 5:**
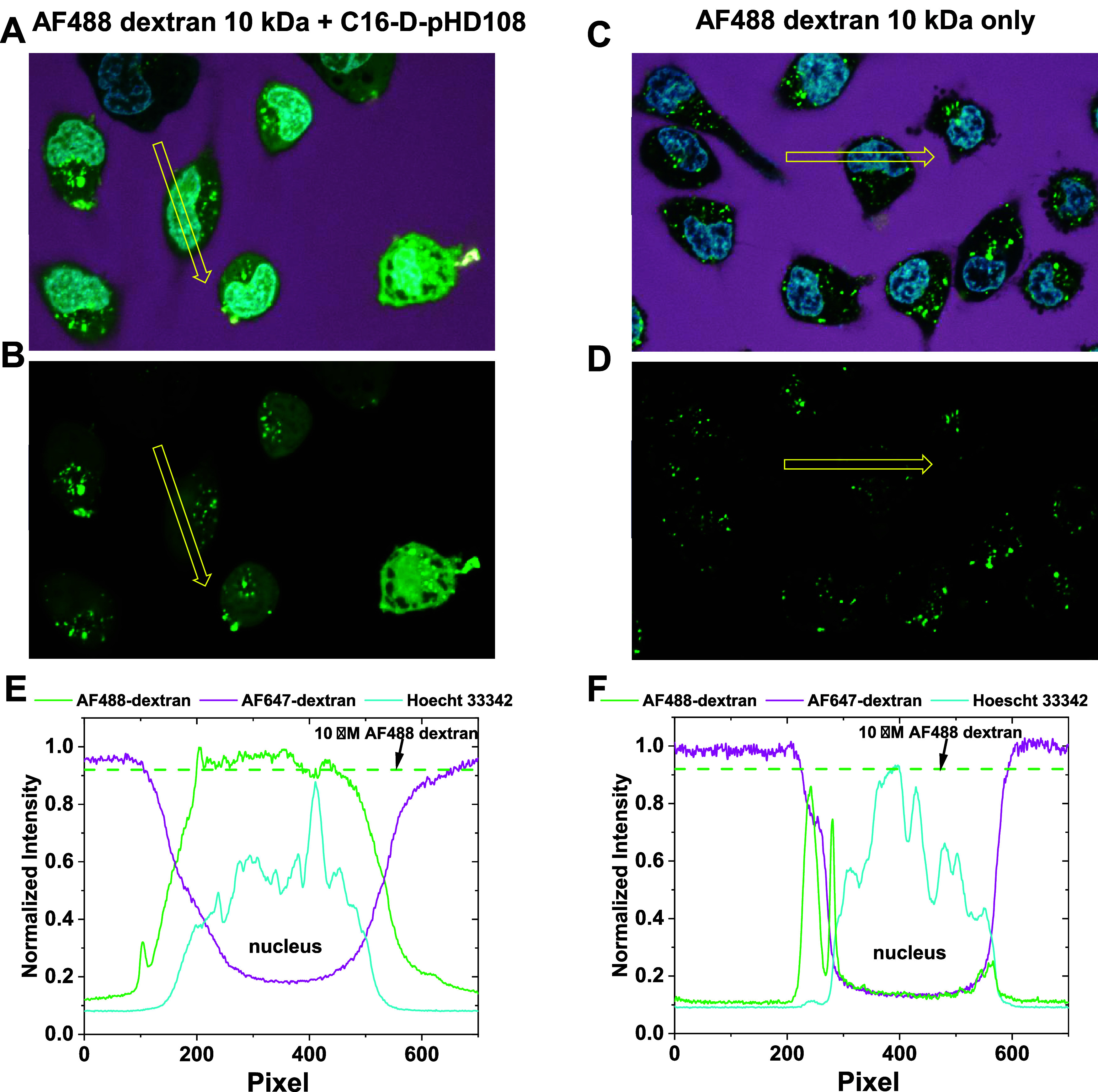
Cytosolic delivery of dye-labeled dextran to cells by C16-d-pHD108. HeLa cells were incubated at 37 °C overnight with 25 μM AF488-dextran 10 kDa with and without 25 μM C16-d-pHD108. Just before imaging, external AF488-dextran (green) was washed off and replaced with AF647-dextran (lavender) to mark the external spaces and cell boundaries. Cells were also treated with Hoechst 33342 (blue) to stain the nuclei. (A) Cells were incubated overnight with AF488-dextran and C16-d-pHD108. (B) The same image as in Panel A, except only the dextran fluorescence, is shown. (C) Cells were incubated overnight with AF488-dextran in the absence of C16-d-pHD108. (D) The same image as in Panel C, except only the dextran fluorescence is shown. (E) Example of normalized intensity measurements across the extracellular spaces, cytosol, and nucleus of the cell indicated by the arrow in panel B. (F) Example of normalized intensity measurements across the extracellular spaces, cytosol, and nucleus of the cells indicated by the arrow in panel D. The intensity of a 10 μM standard solution of AF488-dextran, 40% of the external concentration, is shown as a dashed line in panels (E, F).

In Figure 5, we demonstrate the cytosolic delivery of AF488-dextran 10 kDa to cells by 25 μM C16-d-pHD108 and show how the delivery was measured. HeLa cells were incubated overnight with 10 kDa Alexafluor488-dextran (green), either with or without 25 μM C16-d-pHD108. Confocal microscopy images in Supplemental Figures S1 and S2 verify that both dextran cargos and C16-d-pHD108 enter HeLa cells passively by endocytosis without any significant binding to the cell surface. Just before imaging, cell nuclei were stained with Hoechst 33342 (blue); the external AF488-dextran was washed away, and AF647-dextran (lavender) was added to mark the extracellular spaces and cell boundaries. All cells contained bright individual puncta consistent with endosome-entrapped AF488-dextran after overnight incubation. Some cells also contain perinuclear clusters of bright endosomes. These became more abundant when lower concentrations of dextran were used (see below). When C16-d-pHD108 was present, the nucleus and cytosol were diffusely and uniformly stained with dextran, Figure 5A,B. The diffuse nuclear intensities in Figure 5A,B demonstrate that the dextran was delivered to the cytosol because the nucleus can only be accessed by free dextran in the cytosol, which can diffuse through the nuclear pore complex. The nucleus is not accessible to encapsulated or endosomal dextran, as shown in Figure 5D&E and Figure S1. The fact that the nuclear intensities, from which endosomal structures are absent, match the nominal cytosolic intensities, Figure 5E, also verifies that we are quantitating only endosome-free areas. When C16-d-pHD108 was absent, the endosomes and some perinuclear clusters were present, but the nuclei and cytosol contained little or no free dextran, Figure 5C,D. Thus, in the absence of acylated peptide, no spontaneous endosomal escape occurs even during overnight incubation. Individual intensity scans across single cells, indicated by the yellow arrows, are plotted in Figure 5E,F as examples. To measure cytosolic dextran concentrations, dextran intensity was measured in the external spaces, where it is at background levels and in puncta-free, nuclear/cytosolic areas of each cell. These were compared to the intensities observed in the images of standard solutions. The intensity of a 10 μM solution of AF488, which is 40% of the external concentration used in the experiment, is shown as dashed lines in Figure 5C,D. Intensities of AF488-dextran delivered to the nucleus by 25 μM C16-d-pHD108 in the experiments shown in Figure 5, averaged over multiple cells, and compared to standard solutions are shown in Supplemental Figure S3.

**Figure 6:**
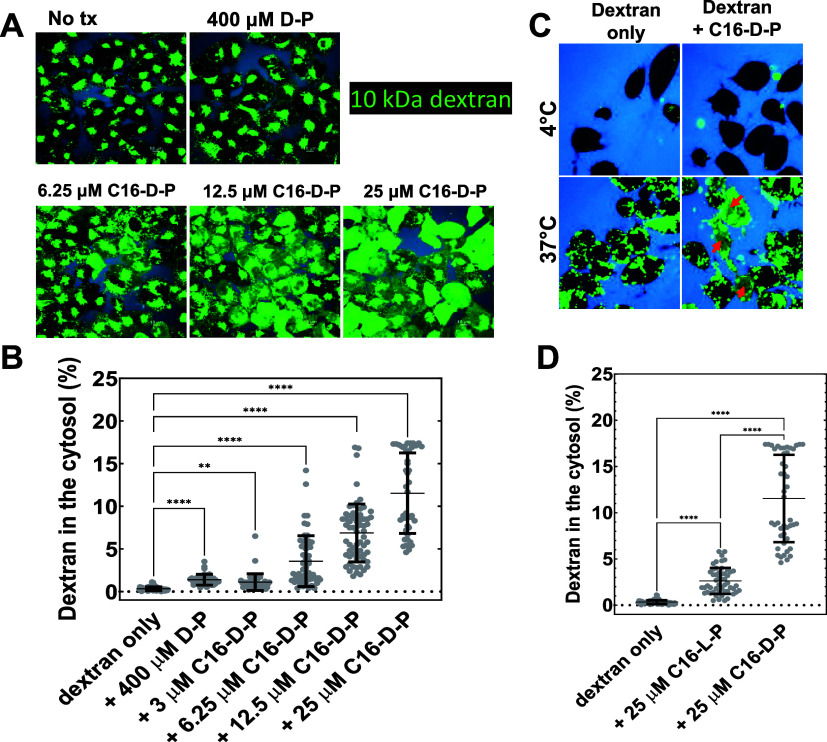
Measurement of cytosolic delivery of AF88-labeled dextran by variants of pHD108. (A) Confocal microscopy images of HeLa cells incubated at 37 °C overnight with 0.1 μM AF488-dextran 10 kDa, plus the pHD108 variants indicated. In all cases, we used confocal images to measure the diffuse fluorescence in the nuclei and cytosol and compare it to that of a standard curve consisting of different concentrations of a reference solution containing AF488-dextran. After incubation, all cells contain bright endosomal puncta that have unreleased dextran, including masses gathered in perinuclear regions. (B) Nuclear and cytosolic fluorescence, identified by diffuse uniform intensity exclusive of bright puncta, was measured in ∼50 individual cells, shown as individual points. In the absence of peptides, cytosolic and nuclear fluorescence is negligible. By one-factor ANOVA, all peptide treatments give statistically significant increases in cytosolic fluorescence, with delivery increasing with the peptide concentration. (C) Cytosolic delivery of dextran at 4 and 37 °C, with and without C16-d-pHD108. (D) Comparison of cytosolic delivery of AF488-dextran by C16-l-pHD108 and C16-d-pHD108, measured as described above for panel B.

In Figure 6, we assessed the delivery of dextran to the cytosol using one high concentration of unmodified d-pHD108 as well as a series of lower concentrations of C16-d-pHD108. These experiments were done using a very low solution concentration of 0.1 μM AF488-dextran 10 kDa to minimize any possible self-quenching in endosomes and to minimize any osmotic effect of the dextran on endosomal stability. HeLa cells were incubated with 0.1 μM AF488-dextran 10 kDa alone or with pHD108 or C16-d-pHD108 overnight. Before imaging, the AF488-dextran was washed off, and Cascade Blue dextran 10 kDa was added to mark the external spaces and cell boundaries. This dextran does not enter cells, confirming that the plasma membranes are not permeabilized, consistent with the lack of toxicity caused by C16-d-pHD108 alone at these concentrations, Figure 4E. Cells were imaged by confocal fluorescence microscopy to distinguish nuclear/cytosolic AF488-dextran from endosome-entrapped dextran and external spaces, as demonstrated in Figure 5. Endosome-entrapped dextran was found in bright individual puncta within the cells, and also in large perinuclear vesicle clusters. Dextran delivered to the cytosol and nucleus, which was the only intensity that we measured in Figure 6A,B, was identified by its diffuse and uniform intensity in the nucleus and cytosol of each cell. In Figure 6E, cytosolic and nuclear concentrations of dextran are expressed as a percent of the external concentration incubated with cells based on comparison to a standard curve of fluorescence intensity versus concentration that was measured separately at the same instrument settings.

Low but statistically significant cytosolic dextran concentrations were found in the cells treated with 400 μM unmodified pHD108 and 3 μM C16-d-pHD108. These measurements agree closely with our conclusion from the PE-III assay that C16-d-pHD108 is about 2 orders of magnitude more potent than unmodified pHD108 at macromolecule delivery to the cytosol. Cytosolic dextran delivery increased significantly as C16-d-pHD108 concentration increased from 3 to 25 μM, Figure 6B. At 25 μM C16-d-pHD108, cytosolic dextran concentrations ranged from 5 to 20% of the applied external concentration, with an average of about 12%. We note that at all concentrations of C16-d-pHD108, a significant amount of internalized dextran remains entrapped in endosomes even at the end of the incubation period.

In Figure 6C, we show that dextran uptake and delivery to the cytosol occur only by endocytosis. In this experiment, cells were incubated either at 4 °C or at 37 °C, with dye-labeled dextran, with or without 25 μM C16-d-pHD108. At 4 °C, little to no dextran was taken up by cells (upper left), and the presence of 25 μM C16-d-pHD108 does not enable any measurable delivery of dextran to the cytosol (upper right). At 37 °C, in the absence of peptide (lower left), dextran was taken up into many endosomes but remained entrapped such that the cytosol remained essentially dextran-free. This result is also shown quantitatively in the peptide-free column of Figure 6B. The only condition that gives rise to cytosolic/nuclear dextran is the incubation of cells at a temperature of 37 °C when both dextran and 25 μM C16-d-pHD108 are present in the lower right part of Figure 6C. In this case *only*, most cells have diffuse nuclear fluorescence, demonstrating that the delivery of dextran to the cytosol occurred. This condition is also shown quantitatively in the rightmost column of Figure 6B. A second dextran, labeled with Cascade Blue added just before imaging, shown in blue false color, demonstrates that the plasma membranes are not permeabilized under these conditions. Finally, we compared the delivery of dextran by C16-l-pHD108 and C16-d-pHD108, Figure 6D, and found that the protease-resistant d-pHD108 was significantly more active than the protease-susceptible l-pHD108. This observation offers additional evidence that the protein delivery mechanism occurs via endocytosis and subsequent acidification, leading to the degradation of the l-amino acid peptide but not the d-amino acid peptides by proteolytic enzymes. The residual delivery activity of C16-l-pHD108 but not l-pHD108 suggests that acylation and nanoparticle formation (see below) may partially protect this peptide from proteolytic degradation.

### Fluorescent Protein Delivery

**Figure 7:**
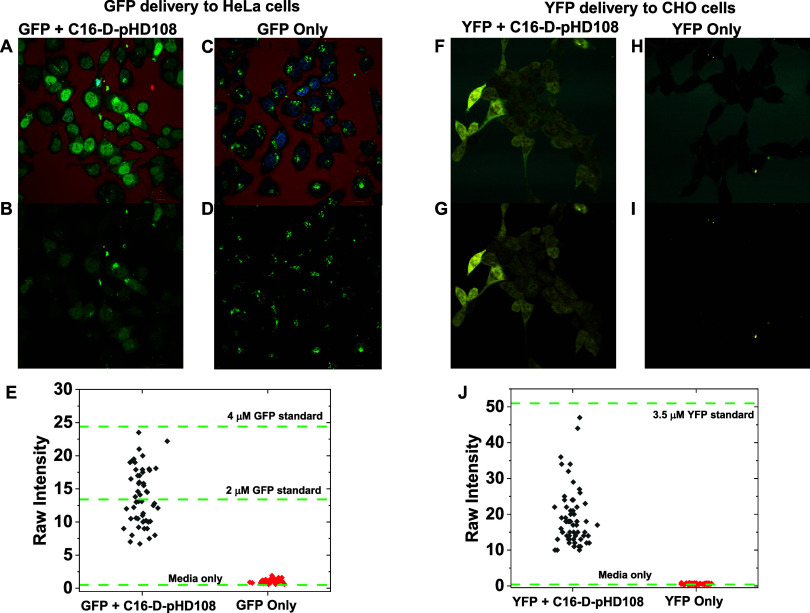
Cytosolic delivery of fluorescent proteins to two different cell lines by C16-d-pHD108. (A–E) HeLa cells were incubated at 37 °C overnight with 10 μM green fluorescent protein (GFP) with and without 25 μM C16-d-pHD108. (F–J): Chinese Hamster Ovary (CHO) cells were incubated overnight with 3.5 μM yellow fluorescent protein (YFP) with or without C16-d-pHD108. (A, B) GFP delivery to HeLa cells was confirmed with C16-d-pHD108. Just before imaging, the external GFP was washed off and replaced with TAMRA-dextran (red) to mark the external spaces and cell boundaries. The nuclei were stained with Hoechst 33342 (blue). Panel A shows all colors and panel B shows only GFP. (C, D) GFP delivery to HeLa cells in experiments identical to panels (A and B), except that C16-d-pHD108 was absent. Panel C shows all colors, and panel D shows only GFP. (E) GFP intensities from the nuclei of the cells in panels (A–D). Dotted lines are the intensities of standard reference solutions of 0, 2, and 4 μM GFP measured under identical conditions. (F, G) YFP delivery to CHO cells was carried out with 25 μM C16-d-pHD108. Just before imaging, the external YFP was washed off and replaced with fluorescent protein mTurquoise (mTurq) (blue) to mark the external spaces and cell boundaries. Panel F shows both colors and panel G shows only YFP. (H, I) YFP delivery to CHO cells in experiments was identical to that of panels (G and F), except that C16-d-pHD108 was absent. Panel H shows both colors and panel I shows only YFP. (J) YFP intensities from the nuclei and cytosol of the cells in panels (F–I). The dotted lines are the intensities of the standard reference solution of media only and 3.5 μM YFP measured under identical conditions.

To validate the dextran delivery experiments, we also delivered two separate fluorescent proteins of ∼27 kDa molecular weight to the cytosol of two different cell lines using C16-d-pHD108 (see Figure 7). HeLa cells were incubated overnight with 10 μM green fluorescent protein (GFP), and Chinese Hamster Ovary (CHO) cells were incubated overnight with 3.5 μM yellow fluorescent protein (YFP), with and without 25 μM C16-d-pHD108. Just before imaging, external GFP and YFP were washed off and replaced with TAMRA-dextran (red) or fluorescent protein mTurquoise (blue), respectively, to mark the cell boundaries and extracellular spaces. Cells that were incubated with either fluorescent protein plus 25 μM peptide contain diffuse nuclear/cytosolic FP staining, Figure 7A,B,F,G. Diffuse nuclear staining that is equal to the cytosolic staining shows that cytosolic delivery has occurred because only free FPs in the cytosol can diffuse into the nucleus. Cells incubated with GFP or YFP, but no peptide, have little nuclear fluorescence, Figure 7C,D,H,I, indicating that no delivery of FP to the cytosol occurred. These cells, especially those incubated with the higher 10 μM concentration of GFP, show endosomes with entrapped FP, just as we observed for dextran delivery. FP intensities measured in cell nuclei were compared to intensities measured in media and standard reference solutions under the same conditions, as shown in Figure 7E,J. With an external concentration of 10 μM, the GFP concentration delivered to cell cytosol by 25 μM C16-d-pHD108 ranges from 1 to 4 μM, with a median of about 2 μM, or 20% of the external concentration. The apparent nuclear concentration of GFP in cells incubated with GFP but no peptide is indistinguishable from the background. With an external concentration of 3.5 μM, the YFP concentration delivered to the cell cytosol ranges from 1 to 3 μM, with a median of about 1.2 μM or 35% of the external concentration. The apparent cytosolic concentration of YFP in cells incubated with YFP but no peptide is indistinguishable from the background. We also performed YFP delivery experiments at a 4 h time point, Supplemental Figure S4. These data show that cytosolic delivery is measurable at 4 h but that the median concentration of cytosolic YFP is about 13% of the external solution, compared to about 35% after overnight incubation. For comparison, we also delivered an (F_ab_)_2_ fragment of an antibody (∼110 kDa) overnight and observed 9% of the external concentration in the cytosol, Figure S5.

### Acylation of pHD108 Drives Nanoparticle Formation

**Figure 8:**
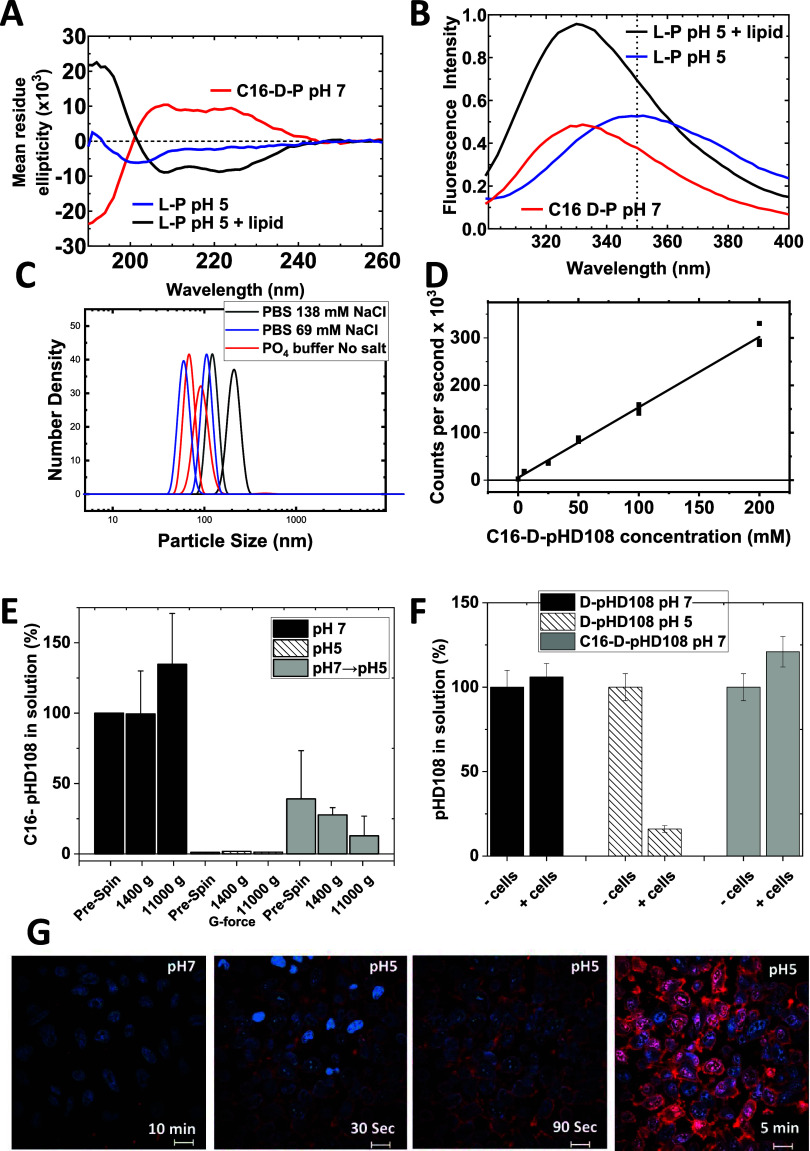
Solution properties and cell binding of pHD108 variants. (A) Circular dichroism spectra of C16-d-pHD108 at pH 7, and of unmodified l-pHD108 at pH 5 with and without liposomes. Spectra were collected at 25 μM peptide in phosphate-buffered saline. (B) Tryptophan fluorescence emission spectra of the same three solutions are characterized in panel A. The dotted line indicates the emission maximum observed for pHD108, which is monomeric and its tryptophan residue is exposed to bulk water. (C) Particle size distribution in individually prepared 25 μM solutions of C16-d-pHD108 measured by quasielastic light scattering on a Malvern Zetasizer nano DLS instrument. Buffers contained phosphate plus 138 mM, 69 mM, or 0 mM added NaCl. (D) Light scattering intensity, in counts per second, is shown as a function of peptide concentration. (E) Solubility of pHD108 and C16-d-pHD108. Peptides were suspended by gentle vortexing for 1 min, followed by bath sonication for 5 min. After at least 1 h of incubation, solutions were sampled by reverse phase HPLC with and without centrifugation at 1400*g* and subsequent centrifugation at 11,000*g*. The peptide remaining in the solution was normalized to an uncentrifuged sample. (F) Cell binding of the pHD108 variants. HeLa cells at 10^6^ cells/ml were suspended in phosphate-buffered saline at pH 7 or pH 5, followed by the addition of peptide dissolved in water. After 1 h of incubation, the cells were pelleted by centrifugation and the peptide remaining in the solution was measured by HPLC. (G) Confocal microscopy images of HeLa cells incubated with 5 μM unlabeled C16-d-D-pHD108 mixed with 1 μM C16-d-pHD108 labeled with TAMRA. All images were collected with the same instrument settings and the same peptide concentrations. In the left panel, cells were incubated with peptide in media at pH 7 for 10 min. The other panels show one field of cells under the same conditions after the solution pH was reduced to pH 5. Cells are also stained with the DNA-binding dye Hoechst 33342.

Even the most hydrophobic of the acylated peptides, C16-d-pHD108, was soluble in PBS and remained in solution at pH 7 for days. Previously, we showed that other acyl-pHD108 variants with chains greater than 6 carbons self-assembled into oligomeric complexes in solution at pH 7.^44^ Here, in Figure 8, we examine the solution behavior of C16-d-pHD108 using tryptophan fluorescence to measure the water exposure of the tryptophan residue at position 19, Figure 1, and using circular dichroism spectroscopy to measure the secondary structure. For controls, we use unmodified pHD108 at pH 5, which switches from a monomeric random coil in solution to a membrane-bound α-helix when liposomes are added.^28−30,44^

In Figure 8A, we show circular dichroism spectra for unmodified pHD108 at pH 5 and for C16-d-pHD108 at pH 7. Unmodified pHD108 has a random coil structure in solution.^45^ When 1 mM phosphatidylcholine liposomes are added, the peptide binds and adopts a secondary structure, predominantly α-helix, as shown by the classical helical minima at 208 and 222 nm and the maximum at ∼195 nm. The d-amino acid C16-d-pHD108, which has a circular dichroism spectrum with the opposite sign as the l-amino acid pHD108, had a predominantly α-helical secondary structure at pH 7 in buffer, indicating that a higher-order structure has formed in buffer.

In Figure 8B, we show tryptophan emission spectra for the same three samples. Unmodified pHD108 in buffer at pH 5 had tryptophan fluorescence emission with an emission maximum at 350 nm, indicating that the Trp residue at position 19 (Figure 1) is mostly exposed to bulk water,^28−30,46^ consistent with a monomeric, unstructured state. When lipid vesicles were added, pHD108 at pH 5 bound strongly and the tryptophan fluorescence was “blue-shifted” to an emission maximum of 332 nm, demonstrating a significantly reduced exposure of the tryptophan to bulk water,^47^ which is known to accompany membrane binding.^28−30,47^ In comparison, even at pH 7, C16-modified pHD108 had a blue-shifted tryptophan fluorescence spectrum with an emission maximum of 330 nm, indicating that the Trp residue is buried in a higher-order structure. Taken together, these results show that C16-modified pHD108 is preassembled in buffer at pH 7 into a structure that drives the adoption of an α-helical secondary structure and greatly reduces the exposure of the tryptophan residue to bulk water.

Based on these results, we hypothesized that palmitoylation drives the formation of stable, micelle-like particles, with the acyl chains forming a central hydrophobic core and the α-helical, amphipathic pHD peptides forming the external surface of the particle. To test this hypothesis, we next studied the C16-d-pHD108 particle size in solution using quasielastic light scattering (QLS). Samples of C16-d-pHD108 were dissolved in PBS with 138 mM NaCl, PBS with 69 mM NaCl, or PO_4_ buffer with no added salt. Most measurements were made at 25 μM peptides, but we tested concentrations between 25 and 200 μM peptides. Buffer without peptides gave a very low scattering intensity and no coherent particle peaks. Peptide samples showed monodisperse distributions that varied somewhat between different sample preparations. Peak maxima were always between 60 and 200 nm, and these particle size distributions were stable up to a week after sample preparation. Salt concentrations of 0, 69, and 138 mM had only small effects on the nanoparticle size. The median particle size for all samples was ∼120 nm. Particle size distributions for 25 μM C16-d-pHD108 are shown in Figure 8C at several salt concentrations for independently prepared samples. In Figure 8D, the light scattering intensity is shown to be a linear function of peptide concentration, with a Y-intercept at zero. This demonstrates that the particle peaks observed in Figure 8C are derived from scattering caused only by the acylated peptide.

We next measured the solubility and stability of C16-d-pHD108 nanoparticles in solution to observe how stability changes with pH. This was done by incubating 50 μM peptide under each experimental condition for 60 min, followed by centrifugation at 1400*g* to remove large aggregates, and by subsequent centrifugation at 11,000*g* to remove intermediate aggregates. Peptide concentration in solution before and after centrifugation was determined by reverse phase HPLC. C16-d-pHD108 remains soluble at pH 7 and is not removed by centrifugation, Figure 8E, indicating that most of the peptide is found in the stable micellar nanoparticles that were detected by DLS, Figure 8C. When we attempted to dissolve C16-d-pHD108 at pH 5, almost none of the peptides went into solution, Figure 8E. When C16-d-pHD108 was initially dissolved at pH 7, where it is soluble, and then the pH was decreased to pH 5, the peptide became insoluble, and little of the initial peptide remained in solution after centrifugation.

To test whether the peptide partitions from the nanoparticles into cell membranes, we measured the binding of pHD108 and C16-d-pHD108 to cells. This was done by equilibrating a peptide solution with and without cells for 30 min, followed by measurement of the peptide remaining in solution using reverse phase HPLC. At pH 7, unmodified pHD108 remains in solution and does not bind to cells, as expected, Figure 8F. At pH 5, unmodified pHD108 remains in solution if no cells are present (also shown in Figure 8E) but binds strongly to cells if they are present. When we incubated C16-d-pHD108 with cells at pH 7, no measurable binding was observed, as shown in Figure 8F. Because peptide precipitation at pH 5 will mimic cell binding in this experiment, we measured cell binding of acylated pHD108 at pH 5 using dye-labeled C16-d-pHD108 and confocal microscopy, Figure 8G. In this experiment, we mixed 1 μM dye-labeled C16-d-pHD108-TAMRA with 5 μM unlabeled C16-d-pHD108 at pH 7 and incubated with cells for 10 min before imaging. The cells were also stained with the DNA staining dye Hoechst 33342. At pH 7, only very faint membrane-associated TAMRA fluorescence was observed after 10 min. However, when the pH was decreased to ∼pH 5, C16-d-pHD108-TAMRA bound immediately to the cell membranes, showing intense membrane staining by 30 s. Cell/membrane staining by C16-d-pHD108-TAMRA continues to grow rapidly as peptides accumulate on the membranes.

Taken together, the data in Figure 8 show that C16-d-pHD108 self-assembles into stable micellar nanoparticles at pH 7. At this pH, C16-d-pHD108 in micellar nanoparticles does not partition measurably from the nanoparticles into cells, and the nanoparticles themselves, which are anionic at pH 7, do not bind to cells. When the pH was decreased, the nanoparticles became unstable, and the acylated peptide bound strongly to cell membranes if membranes were present or precipitated out of solution if membranes were absent.

## Discussion

In this work, we showed that the synthetically evolved, pH-responsive, nanopore-forming peptide pHD108 can deliver macromolecular cargo to the cytosol of multiple cell lines. The potency of delivery is increased by about 2 orders of magnitude when the peptide is N-palmitoylated. Acylated pHD108 molecules self-assemble into stable anionic micellar nanoparticles at pH 7. When cells are incubated with these nanoparticles along with a small toxin protein (PE-III), fluorescent proteins, or a protein-sized dye-labeled dextran cargo, delivery of the macromolecule to the cell cytosol occurs by a mechanism that requires endocytosis of cargo and acyl-pHD108 nanoparticles, followed by acidification-triggered nanoparticle destabilization that drives nanopore formation in the endosomal membrane. The protein delivered to the cytosol can reach μM concentrations and can be a significant fraction of the external concentration. Delivery occurs similarly in multiple cell types.

Several lines of evidence show that the delivery mechanism in live cells is solely via endocytosis. First, no macromolecule delivery occurs at 4 °C when endocytosis does not occur. We note in published studies that some membrane-acting peptides remain active at low temperatures and can directly translocate across^12,13,40^ or permeabilize the plasma membrane. Thus, the physical state of the plasma membrane at 4 °C does not completely preclude the direct action of peptides. Yet, the pHD peptides are inactive at low temperatures. Second, l-amino acid pHD peptides are much less active in our cellular delivery assays compared to protease-resistant d-amino acid peptides due to their sensitivity to acid-induced endosomal exo- and endoproteinases. This is consistent with the requirement that some acidification must occur to activate the acylated peptide nanoparticles. In contrast, cytolytic or plasma membrane-translocating peptides are similarly active as d- or as l-amino acid peptides because they do not require endocytosis. The fact that C16-l-pHD108 has some residual delivery activity, while unmodified l-pHD108 does not, suggests that palmitoylation and nanoparticle formation are somewhat protective against proteolysis. Since we show that enzymatically active PE-III and fluorescent proteins are effectively delivered to the cytosol, delivery likely occurs early into the endosome-lysosome maturation process as the PE-III enzyme and fluorescent proteins would be inactivated by endosomal protease activity if they remained in endosomes for too long. However, we note that PE-III may have evolved some resistance to proteolysis at low pH.^37^ Furthermore, the more proteolysis-susceptible R494A mutant has the same activity as the wild type, adding support for the conclusion of early endosomolysis as the step where delivery occurs.^37^

We note that we do not have direct evidence of nanopore formation in the endosomal membrane. However, we infer nanopore formation from multiple lines of evidence presented here and published previously. For example, using atomic force microscopy, we have direct evidence of nanopore formation by pHD108 at pH 5 in synthetic phosphatidylcholine membranes^23,30,48^ and also have indirect evidence of nanopores in the same synthetic membranes from studies of leakage of small molecules and dextrans.^23,27−30,49^ Similarly, leakage studies demonstrate that nanopore formation in synthetic bilayers is not inhibited by acylation.^44^ We show here that nanoparticles of C16-pHD108 disassemble at pH 5 and that the peptide quickly binds to the membranes. Almost certainly, the same process occurs in the endosome upon acidification. Finally, the effective delivery of the 25 kDa PE-III, two 27 kDa fluorescent proteins, and a 10 kDa dextran into the cytosol at a relatively low peptide concentration suggests that macromolecule-sized pores are formed, at least transiently, in the endosomal membranes.

The strategy of lipidating peptides has previously been used to increase membrane-active peptide potency.^50,51^ The resulting activity boost given through lipidation is generally attributed to increasing the hydrophobicity of the peptide, a strategy that has long been a useful tool in basic research as well as in drug development.^52,53^ Trichogin, for example, is an antimicrobial peptide drug that has a C8 chain conjugated to the N-terminus, which increases monomeric binding to membranes.^54^ Daptomycin is a natural antimicrobial peptide with a lipid-like tail that inserts into bacterial membranes and disrupts cell wall synthesis, ultimately killing the bacteria.^55^ GALA, a pH-sensitive pore-forming peptide that forms only small pores^27^ has higher permeabilizing activity at low pH when modified by a C12 chain.^56^ Similarly, the antimicrobial tripeptide AKK shows much increased bactericidal activity with palmitoylation.^57^*In vivo*, lipidated peptides often show much better pharmacodynamics and pharmacokinetics as compared to their nonlipidated counterparts.^52^ However, the behavior of lipidated peptides is complex. In the case of trichogen, the lipopeptide monomers did indeed have a higher affinity for membranes compared to a nonlipidated peptide, but at the same time, lipopeptide micelle formation occurs, which reduces the amount that can react with membranes. These opposing forces resulted in similar membrane permeabilizing activity in synthetic membranes for lipidated and nonlipidated peptides. GALA, with a C18 chain, similarly shows a micelle formation. However, GALA has switched pH-sensitive activity compared to the pHD peptides, with higher permeabilizing activity in synthetic vesicles at pH 7.5 compared to pH 5.5.

For the pHD peptides, palmitoylation did not result in increased interactions with cell membranes at pH 7. Instead, C16-pHD108 is self-assembled into stable micellar nanoparticles in which the pHD peptides have a highly α-helical secondary structure and do not interact strongly with cells. Nanoparticle formation is correlated with the potency of cargo delivery to cells as PE-III delivery increased dramatically when the acyl chain length was increased from C4/C6, where nanoparticle assembly is low,^44^ to C8/C12/C16, where nanoparticle assembly is high. Importantly, the nonmicellar C4 and C6 acylated pHD108 variants were the only pHD108 variants that showed some cytotoxicity at high concentrations.

In a recent molecular dynamics study,^34^ we described how the highly polar and anionic surfaces of amphipathic helical peptides that are very closely related to the pHD peptides can drive the self-assembly of large membrane-spanning nanopores. The nanopores are stable because the anionic and polar side chains participate in a highly cooperative membrane-spanning network of direct and water-bridged hydrogen bonds, which is made possible by the amphipathic helical structure of the peptides. Deprotonated, anionic glutamate residues are critical elements of this stabilizing H-bond network.^34^ We speculate that the micellar nanoparticles formed by C16-pHD108 are stabilized by a similar interaction network, with the palmitate chains forming an internal hydrophobic core layer and the peptides forming a self-stabilizing, water-exposed outer layer. The geometry of the nanoparticles is currently unknown and could be rod-like, disk-like, or spheroidal. This manner of nanoparticle stabilization could explain why the acyl peptide does not partition from the nanoparticles into the cell membranes at pH 7. At pH 5, we hypothesize that protonation of some glutamate residues in C16-pHD108 destabilizes the H-bond network, which destabilizes the nanoparticles, resulting in the partitioning of the acyl peptides into membranes, where they can self-assemble into nanopores.

### Study Limitations

The findings of this work should be interpreted in the context of the specific assays used to measure the macromolecule delivery. Foremost among the limitations is the use of the PE-III enzyme as a macromolecular test cargo. Cytosolic PE-III causes apoptosis, the signal we use to measure its delivery, after just one or a few molecules successfully reach the cytosol.^36^ Thus, the delivery of PE-III is amplified and is biased toward reporting success. To partially mitigate this effect, we use 40 nM PE-III. At this low concentration, an ideal spherical endosome of 500 nm diameter will contain, on average, just over one passively entrapped PE-III molecule, while a 200 nm endosome will contain an average of 0.1 PE-III molecules (see the Supporting Information). As a rough estimate, the peptide-induced release of contents from 3 to about 40 endosomes can account for our observations of successful PE-III delivery by pHD108. In terms of future applications, PE-III delivery by C16-d-pHD108 may be relevant to targeted enzyme delivery, but the delivery of an inhibitor of protein–protein interactions will require a higher concentration of cargo delivery to the cytosol. Many protein members of signaling networks have copy numbers ranging from 1000 to 50,000 per cell,^58^ which is equivalent to concentrations in the nanomolar range. The dextran and fluorescent protein delivery experiments, which lack amplification, are more realistic mimics of the delivery of relevant concentrations of such inhibitory macromolecules. Here, we show that delivery by C16-d-pHD108 results in the internal macromolecule concentrations that are substantial fractions of the external solution concentration and can readily reach the μM range, although such degree of delivery requires higher concentrations of peptide. Finally, the efficacy of cargo delivery in the system, as described here, is currently limited by the fact that neither the acyl-peptide nanoparticle nor the macromolecular cargos contain any specific targeting moieties directing them to the cell surface or to specific uptake pathways. This limitation highlights that targeting is the obvious next step in the optimization of acylated pHD peptides for macromolecular cargo delivery.

### Conclusions

The palmitoylated peptide C16-d-pHD108 self-assembles into pH-responsive nanoparticles at neutral pH, which, after being passively taken up into endosomes, enable the delivery of endocytosed macromolecules to the cytosol of human cells. This occurs by the pH-driven destabilization of the nanoparticles, which leads to the formation of nanopores in endosomal membranes.

## Materials and Methods

### Peptides

pHD108 variants were synthesized by Biosynthesis, inc. All peptides were ≥95% pure by reverse phase HPLC and had the correct mass by MALDI mass spec. Peptides were stored at −20 °C as lyophilized powders. Stock solutions were made at 1–3 mM in 0.025% glacial acetic acid, and concentrations were determined by optical absorbance at 280 nm, using an extinction coefficient of 5550 M^–1^cm^–1^.

### pHD108 Variants

For C-terminal conjugations, d- or l- pHD108 with a C-terminal GC dipeptide (pHD108-GC) was modified on the cysteine sulfhydryl group by the formation of a disulfide bond with a thioacyl group. In order, we added (by volume) 40% dimethyl sulfoxide (DMSO), 10% of 1% N,N-diisopropylethylamine (DIPEA) in water, 10% of 25 mM stock of one of the following: 1-butanethiol (C4), 1-hexanethiol (C6), 1-octanethiol (C8), 1-dodecanethiol (C12), or 1-hexadecanethiol (C16) prepared in methanol just prior to the conjugation, and 40% of 500 μM d-pHD108-GC from a 1.25 mM stock in 0.025% acetic acid (AcOH). The reaction mixture was vortexed briefly and incubated for 2 h at 56 °C in a water bath. The product was purified by high-performance liquid chromatography (HPLC) using a Macherey-Nagel C2 reverse phase column developed in water and acetonitrile, each with 0.1% trifluoroacetic acid. All expected masses were verified with matrix-assisted laser desorption/ionization-time-of-flight (MALDI-TOF) mass spectrometry.

For N-terminal conjugations, d- or l- pHD108 were modified on the amino terminus using fatty acid anhydrides. In order, by volume, we added 61.3% dimethylformamide (DMF), 2% DIPEA, 16.7% of 500 μM pHD108 from 3 mM stock solution in 0.025% AcOH, and 20% of 10 mM of one of the following: hexanoic anhydride (C6), n-octanoic anhydride (C8), or dodecanoic anhydride (C12), prepared immediately prior in methanol, except for palmitic anhydride (C16), which was prepared similarly in chloroform. The reaction mixture was vortexed briefly and reacted as follows: hexanoic anhydride was reacted for 30 min at room temperature; n-octanoic anhydride was reacted for 2 h at room temperature; dodecanoic anhydride was reacted for 30 min at 57 °C in a water bath; and palmitic anhydride was reacted for 30 min at 50 °C. Purification was performed with a Machere-Nagel reverse phase C2 column developed in water and acetonitrile, each with 0.1% trifluoroacetic acid. All expected masses were verified with matrix-assisted laser desorption/ionization-time-of-flight (MALDI-TOF) mass spectrometry.

### TAMRA-Labeled C16-d-pHD108

To create C16-d-pHD108-TAMRA, we used a pHD108 sequence with a glycine-cysteine dipeptide added to the C-terminus. The peptide was dissolved in dimethylformamide and added to a 3-fold molar excess of TAMRA-maleimide to react with the sulfhydryl group. After two h of reaction, a 5-fold excess of palmitate-succinamidyl ester was added along with 1% v/v diisopropylethylamine to react with the N-terminal amino group. After an overnight reaction, we added 10% water to the sample and purified the acylated and TAMRA-labeled conjugate by HPLC with a Macherey-Nagel C2 reverse phase column.

### PE-III Delivery Assay

Fifteen thousand HeLa cells were seeded in a clear bottom, black-walled 96-well plate overnight at 37 °C with 5% CO_2_ in complete media (DMEM + phenol red +10% FBS + antimycotic/antibiotic + nonessential amino acids). Cells were then washed once in PBS and treated with PE-III and peptide in serum-free media without phenol red, with a 50 μL total volume. After a 2-day incubation with treatment, 50 μL of 10% Alamar blue reagent diluted in serum-free media was added directly to each well (5% Alamar blue final) and incubated for 1.5 h at 37 °C. The plate was then read at 550 equiv/590 em on a Biotek plate reader. Untreated cells were used as a negative control, and MelP5-treated cells served as a positive control.

### Dextran Delivery

For dextran delivery, HeLa cells were incubated at 37 °C overnight in the presence of AF488-dextran (10 kDa) with and without C16-d-pHD108. After overnight incubation, external AF488-dextran (green) was washed off and replaced with Cascade Blue dextran (blue) or AF647-dextran (lavender) just before imaging to mark the external spaces and cell boundaries. Cells were also treated with Hoechst 33342 (blue) to stain the nuclei. Quantitation was performed by generating a standard curve from microscopy images of solutions of AF488-dextran of known concentrations. For quantification, we noted the intensity of the cell nuclei. Multiple small circular areas of interest of a preset size were selected in cells in areas that were in the nucleus or had no obvious puncta. The same was done for the standard curve images. The fluorescence of each point of interest was quantified using the Nikon Elements software, and the concentration was determined using a standard curve and expressed as a percent of the initial incubating concentration.

### Protein Delivery

For fluorescent protein or fluorescein-(F_ab_)_2_ fragment delivery, HeLa cells were incubated at 37 °C overnight in the presence of GFP with and without C16-d-pHD108 and CHO cells were incubated at 37 °C overnight with YFP in the presence and absence of C16-d-pHD108. Just before imaging, the external GFP was washed off and replaced with TAMRA-dextran. External YFP or fluorescein-(F_ab_)_2_ were washed off and replaced with mTurquoise (blue) to mark the external spaces and cell boundaries. Quantitation of delivered protein was performed by comparing measured intensities from microscopy images of standard protein solutions of known concentrations.

### Cell Binding

HeLa or RAW 264.7 cells were trypsinized, washed, and counted. Cells were concentrated in a minimal amount of PBS. To measure binding, 25 μL of cells at a known count were transferred to tubes and 25 μL of peptide in PBS was added for a final peptide concentration of 20 μM. Samples were incubated for 30 min with gentle agitation at room temperature. Cells were pelleted in a swinging bucket centrifuge, and 30 μL of supernatant was subjected to HPLC to measure the peptide remaining in the solution. A sample without cells but treated identically was used as a negative control for binding to normalize the recovered peptide.

### Dynamic Light Scattering

Dynamic light scattering was performed by using a Malvern Zetasizer Nano ZS90 DLS instrument. After HPLC purification, 10 nmol aliquots of C16-d-pHD108 were dried in silanized Eppendorf tubes. Peptide readily dissolved in PBS buffer and other aqueous buffers and remained stable in solution. For DLS samples, C16-d-pHD108 was dissolved in PBS with 138 mM NaCl, in PBS with 69 mM NaCl (50% of normal), or in a PO_4_ buffer with no added salt. Most measurements were made at 25 μM peptide, but we tested concentrations between 25 and 200 μM peptide. Prior to DLS measurements, samples were sonicated and then centrifuged for 5 min at 11,000*g* to remove particulate contaminants. No significant amount of peptide was lost during this step (see the text). The supernatant was placed in a cuvette that had been washed twice with a similarly centrifuged buffer before three data sets were collected and averaged.

### Measurement of Peptide Solubility

Dried peptides were reconstituted in 50 μL of PBS at pH 7 or at pH 5. The solutions were bath-sonicated briefly and incubated at room temperature for 30 min. Three aliquots of each sample were removed for analysis: one before centrifugation, one after centrifugation for 5 min at 1400*g*, and one after centrifugation for 10 min at 11,000*g*. These aliquots were diluted and analyzed by reverse phase HPLC. The integrated peak area of the tryptophan fluorescence peak was determined for each sample.

### Circular Dichroism Spectroscopy

CD spectra were collected using a Jasco J-810 spectrophotometer, flushed with N_2_. Scans were at 20 nm/sec, 3 accumulations, and samples were at room temperature. The quartz cuvette path length was 0.1 cm. Mean residue ellipticity (MRE) was then calculated as MRE = ε/(*Cn*) where ε is ellipticity, *C* is the molar concentration of peptide, and *n* is the number of residues.

## Supporting Information Available

Confocal microscopy images of uptake and delivery; quantitation of protein delivery; quantitation of antibody fragment delivery; model calculation for uptake and delivery (PDF)
