# Supplementary material for: pH-Responsive Peptide Nanoparticles Deliver Macromolecules to Cells via Endosomal Membrane Nanoporation
Source: ACS Nano. 2024 Dec 9;18(50):33922–36. doi: 10.1021/acsnano.4c07525 (PMC11656837; doi:10.1021/acsnano.4c07525)
Supplement: Supplementary file 1 — nn4c07525_si_001.pdf [file nn4c07525_si_001.pdf]

Supplemental information

# pH-Responsive Peptide Nanoparticles Deliver Macromolecules to Cells via Endosomal Membrane Nanoporation

Eric Wu[1], Ains Ellis[1], Keynon Bell[2,3], Daniel L. Moss[1], Samuel J. Landry[1], Kalina Hristova[3,4], William C. Wimley[*,1]

[*]Corresponding author. Email: wwimley@tulane.edu

[1]Department of Biochemistry and Molecular Biology, Tulane University School of Medicine, New Orleans, LA, 70112, USA

[2]Chemistry-Biology Interface Program, Johns Hopkins University, Baltimore, Maryland, 21218, USA

[3]Institute for NanoBioTechnology, Johns Hopkins University, Baltimore, Maryland 21218, USA

[4]Department of Materials Science and Engineering, Whiting School of Engineering, Johns Hopkins University, Baltimore MD, 21218, USA

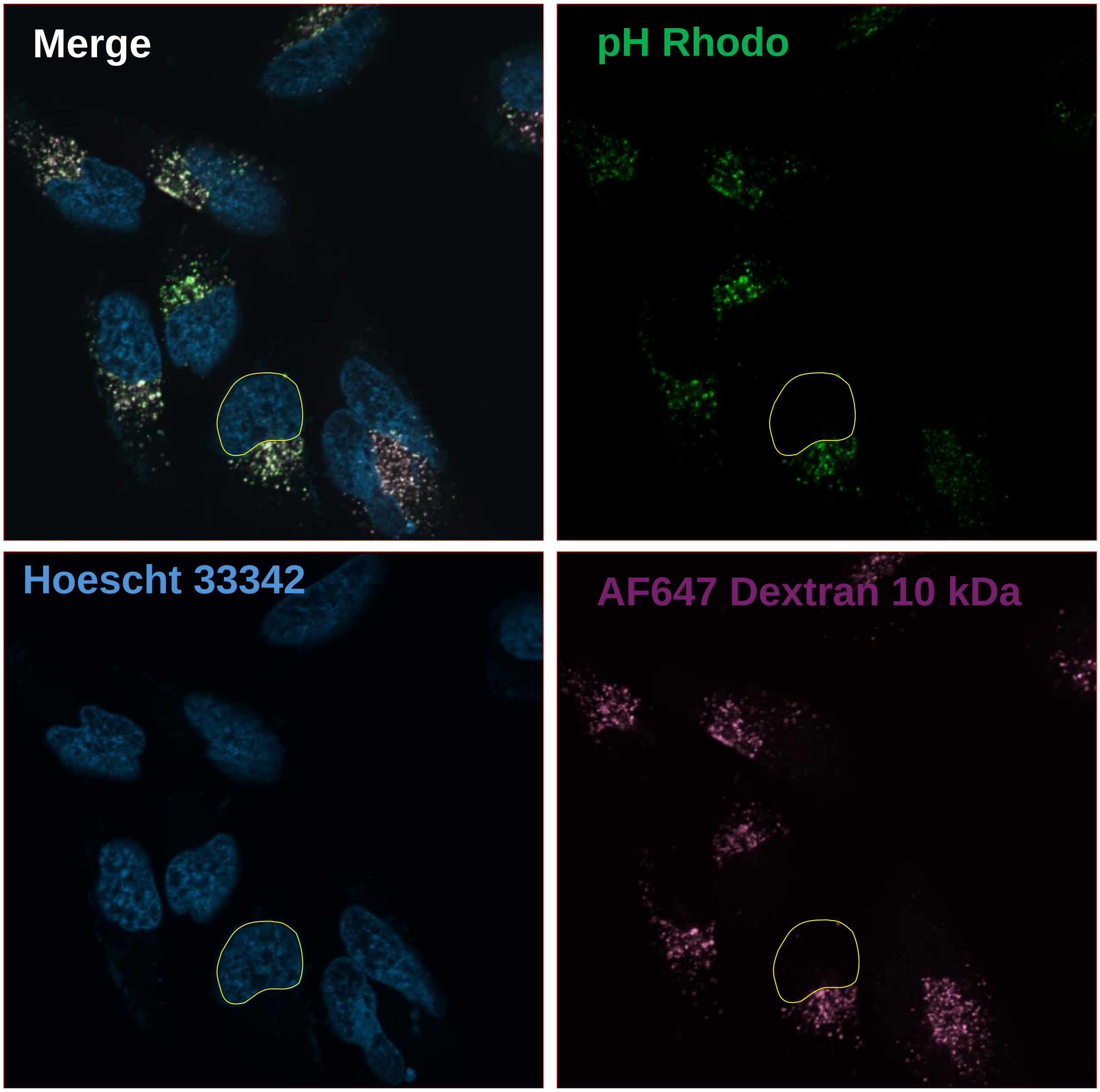


**Figure S1.** Uptake of dye-labelled dextran cargo into HeLa cells is by endocytosis. HeLa cells were incubated for four hours at 37°C with Alexafluor647 Dextran 10 kDa (lavender) and pHrhodo green, an acid pH-responsive endocytosis marker. No pHD108 peptide was present. Cell nuclei were stained with Hoechst 33342 (blue) before imaging. The perimeter of one example cell nucleus is denoted by a yellow line for reference. The dye labelled dextran and endocytosis marker colocalize in puncta observed in the cell cytosol. No fluorescence of either probe is found inside the nucleus, indicating that none of the dyes have entered the cytosol in this experiment. No interaction with the cell surface is detectable. Uptake of a dextran cargo is by passive endocytosis.

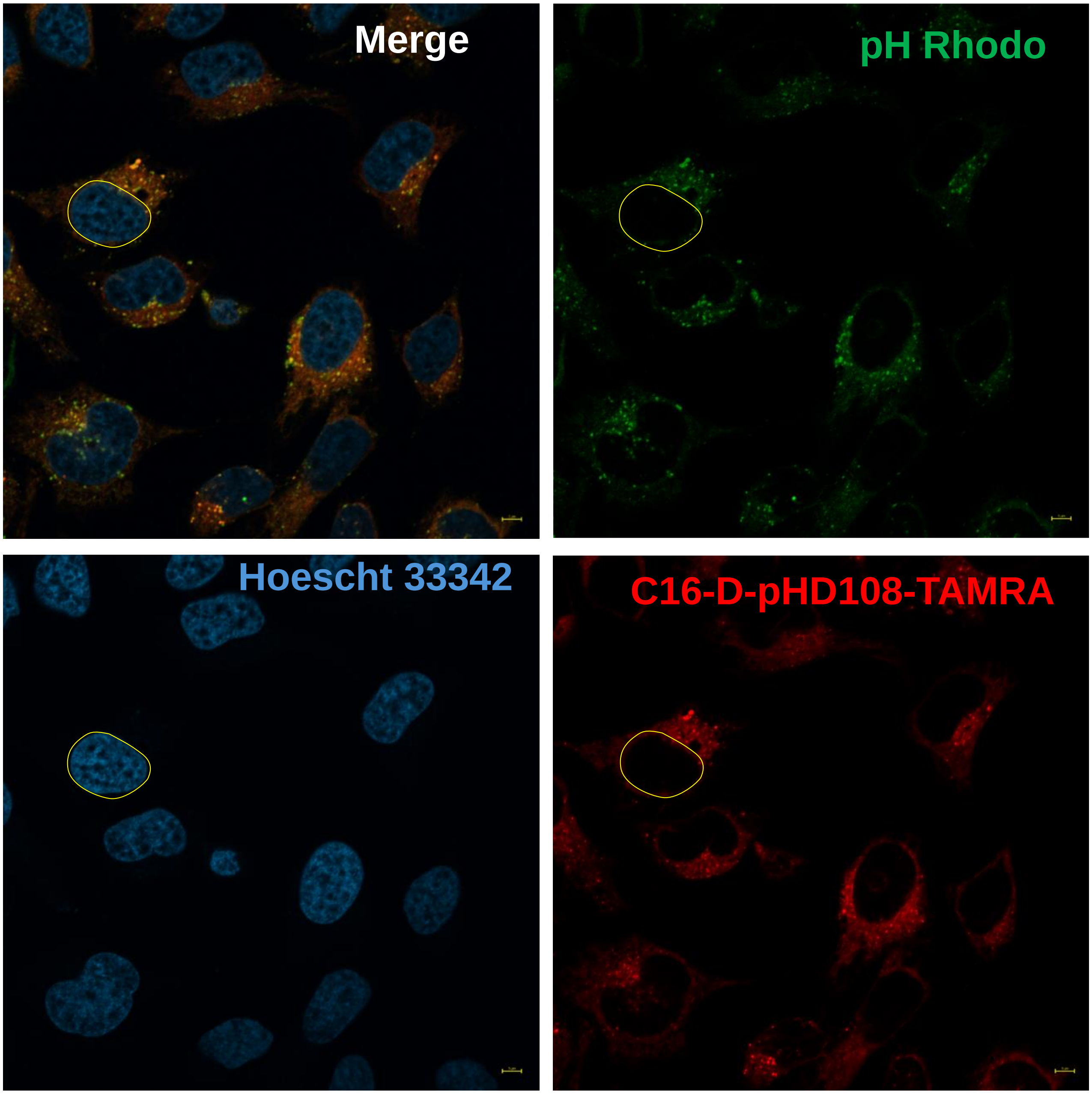


**Figure S2.** Uptake of C16-D-pHD108-TAMRA into HeLa cells is by endocytosis. HeLa cells were incubated for 30 min at 37°C with 1 μM C16-D-pHD108-TAMRA (red) along with pHrhodo green, an acid pH-responsive endocytosis marker. A low concentration of pHrhodo green of 30 μg/ml was used to reduce interference with TAMRA fluorescence. Cell nuclei were stained with Hoechst 33342 (blue) before imaging. The perimeter of one example cell nucleus is denoted by a yellow line for reference. The dye labelled peptide and endocytosis marker colocalize in puncta observed in the cell cytosol. There is also a diffuse TAMRA fluorescence in the cytosol that does not enter the nucleus. No interaction with the cell surface is detectable. Uptake of the peptide is by passive endocytosis.

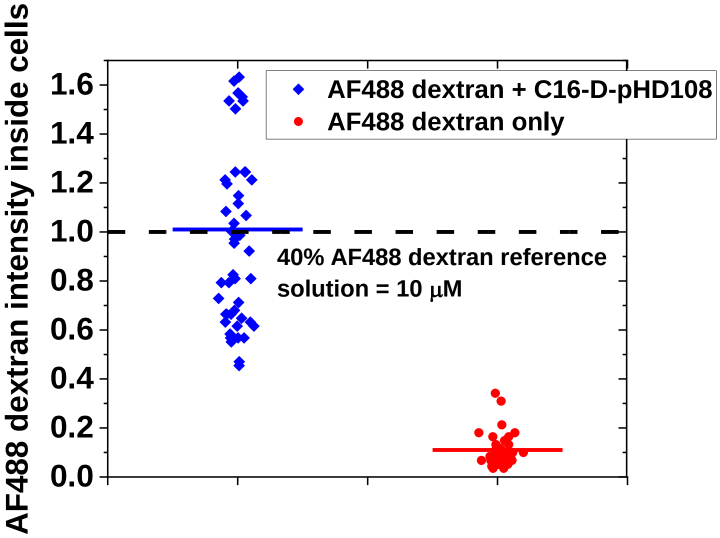


**Figure S3.** Quantitation of dextran delivery to cells by C16-D-pHD108. **A.** HeLa cells were incubated overnight with 25 μM AF488-dextran with or without 25 μM C16-D-pHD108. After overnight incubation, confocal microscopy was used to image the cells after washing away external AF488 dextran, **Fig. 5** main manuscript. Intensities of nuclear spaces in raw images of ~50 cells were measured using imageJ, and are compared to the intensity of a reference solution corresponding to 40% of the external concentration, measured under the same conditions.

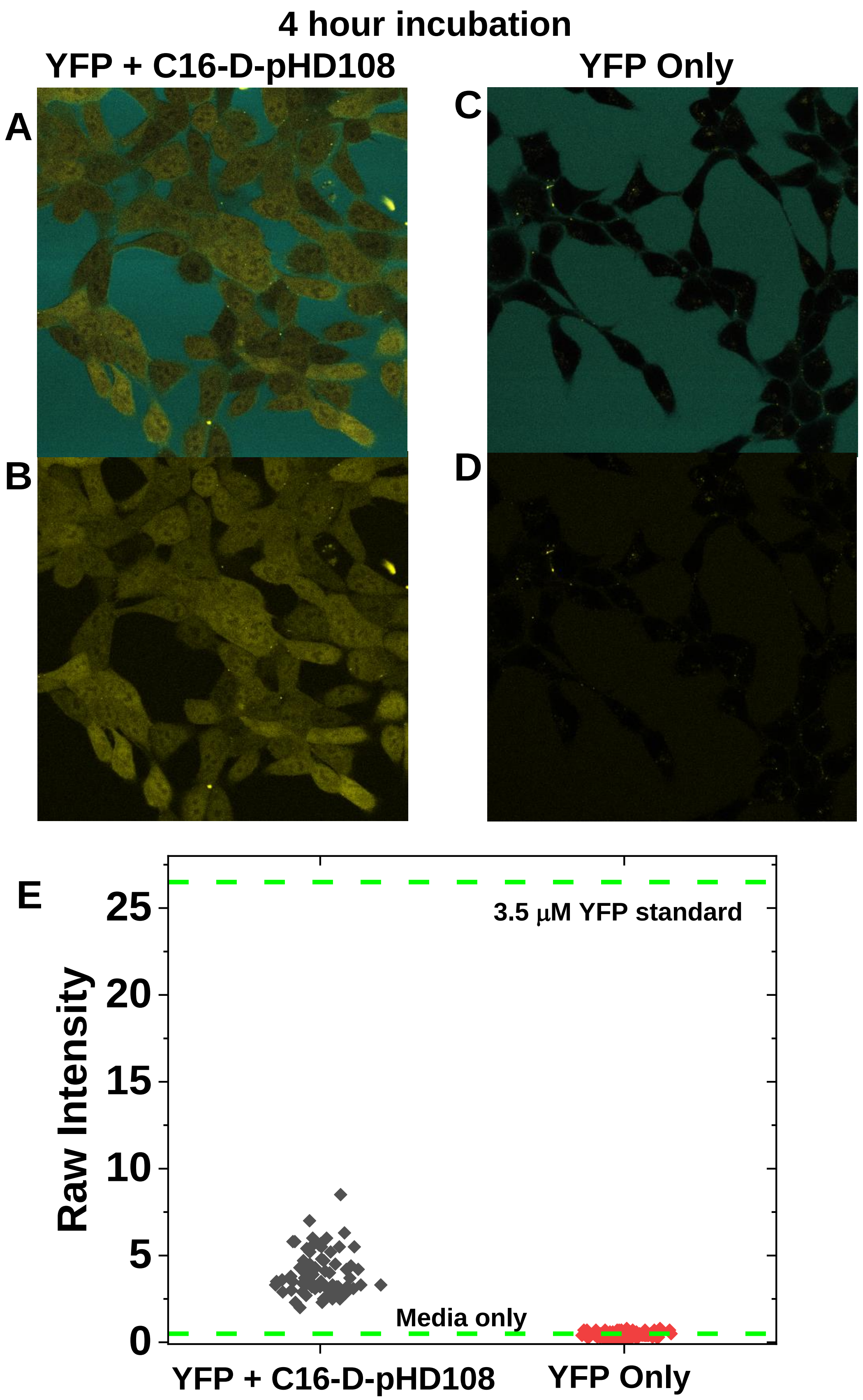
4 hour incubation
YFP + C16-D-pHD108
YFP Only
A
B
C
D
E
Raw Intensity
25
20
15
10
5
0
3.5 μM YFP standard
Media only
YFP + C16-D-pHD108
YFP Only

**Figure S4.** YFP delivery to CHO cells with C16-D-pHD108 after a 4 hour incubation. Chinese Hamster Ovary (CHO) cells were incubated at 37°C for 4 hours with 3.5 µM yellow fluorescent protein (YFP) with or without 25 µM C16-D-pHD108. Just before imaging, the external YFP was washed off and replaced with the fluorescent protein mTurquoise (mTurq) (blue) to mark the external spaces and cell boundaries. **A,B**: Images of cells incubated with YFP and C16-D-pHD108. Panel A shows both colors and panel B shows only YFP. **C,D**: YFP delivery to CHO cells in the absence of C16-D-pHD108 was absent. Panel C shows both colors and panel D shows only YFP. **E.** YFP intensities from the nuclei and cytosol of the cells in panels A,B and C,D. The dotted lines are the intensities of standard reference solution of media only and 3.5 µM YFP measured at the same time under identical conditions. In the presence of peptide, the cytosolic/nuclear intensity of YFP was about 13% of the external solution after 4 hours. In the absence of C16-D-pHD108 cytosolic/nuclear delivery of YFP was absent.

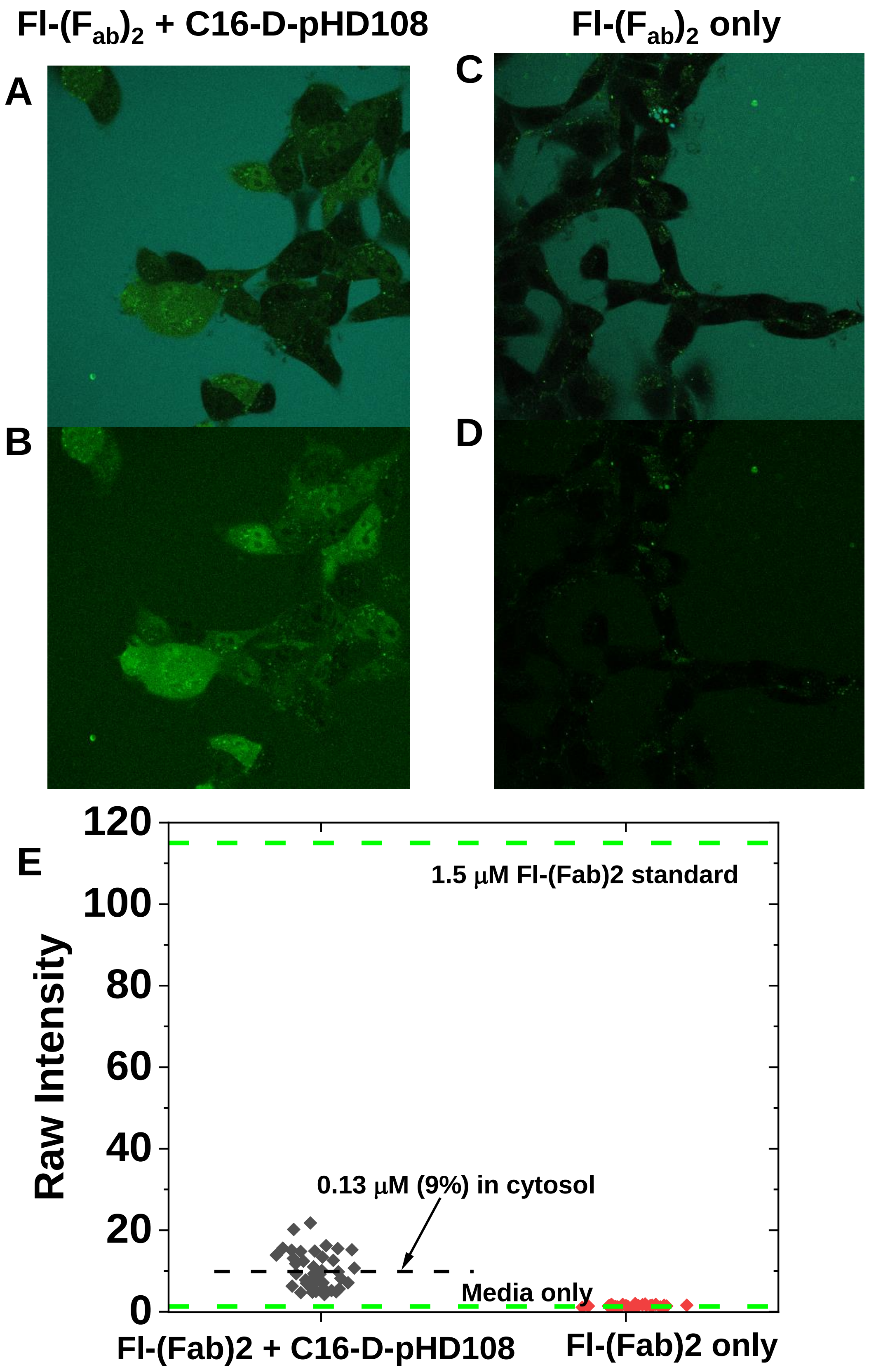

Fl-(Fab)2 + C16-D-pHD108
Fl-(Fab)2 only
A
B
C
D
E
Raw Intensity
120
100
80
60
40
20
0
1.5 μM Fl-(Fab)2 standard
0.13 μM (9%) in cytosol
Media only
Fl-(Fab)2 + C16-D-pHD108
Fl-(Fab)2 only

**Figure S5.** Delivery of a 110 kDa $(F_{ab})_2$ fragment to CHO cells with C16-D-pHD108 after overnight incubation. Chinese Hamster Ovary (CHO) cells were incubated at 37°C with 1.5 μM fluorescein-labelled $(F_{ab})_2$ (green) with or without 25 μM C16-D-pHD108. Just before imaging, the external antibody $(F_{ab})_2$ was washed off and replaced with the fluorescent protein mTurquoise (mTurq) (blue) to mark the external spaces and cell boundaries. **A,B**: Images of cells incubated with Fl-$(F_{ab})_2$ and C16-D-pHD108. Panel A shows both colors and panel B shows only Fl-$(F_{ab})_2$. **C,D**: Images of cells incubated with Fl-$(F_{ab})_2$ without C16-D-pHD108. Panel C shows both colors and panel D shows only Fl-$(F_{ab})_2$. **E.** Fl-$(F_{ab})_2$ intensities from the nuclei and cytosol of the cells in panels A,B and C,D. The dotted lines are the intensities of standard reference solution of media only and 1.5 μM Fl-$(F_{ab})_2$ measured at the same time under identical conditions. In the presence of peptide, the cytosolic/nuclear intensity was about 9% of the external solution. In the absence of C16-D-pHD108 cytosolic/nuclear delivery of Fl-$(F_{ab})_2$ was absent.

## Calculation of the minimal effect required for PE-III assay positive results

### How many endosomes would need to release their contents to explain the PE-III assay results?

### Size of an endosome

Endosome sizes vary widely in size from 50 nm to 1000 nm[1] and they include structures that are spherical or tubular. For example, clathrin-coated endocytic vesicles are 100-200 nm diameter and caveolin-dependent vesicles are 60-80 nm diameter, while macropinocytotic vesicles can be 200-2000 nm in diameter. For these calculations, we will assume spherical endosomes of 500 nm and 200 nm inner diameters.

### Volume of an endosome

An endosome with a diameter of 500 nm has a radius of $2.5 \times 10^{-7}$ m, which gives a volume of $6.5 \times 10^{-17}$ L.

An endosome with a diameter of 200 nm has a radius of $1 \times 10^{-7}$ m, which gives a volume of $4.2 \times 10^{-18}$ L.

### PE-III per endosome.

We use 40 nM PE-III in our delivery assays. This is equivalent to $2.4 \times 10^{16}$ molecules per liter. Thus, a 500 nm diameter endosome with a volume of $6.5 \times 10^{-17}$ l will contain an average of 1.5 PE-III molecules each, distributed in a Poisson distribution, assuming simple passive entrapment. A 200 nm diameter endosome with a volume of $4.2 \times 10^{-18}$ l will contain an average of 0.1 PE-III molecules each, distributed in a Poisson distribution, assuming simple passive entrapment.

### PE-III per cell.

For this calculation, we assume that that 100% of our PE-III molecules are enzymatically active and that a single PE-III molecule can induce apoptosis in a metabolically active cell[2]. If PE-III molecules are distributed randomly, populations will be described by the Poisson equation. At 40 nm PE-II and 10 μM C16-pHD108, we observe that >98% of all cells undergo apoptosis, thus the probability of having zero PE-III per cell must be less than 0.02. The average number of delivered PE-III per cell that would ensure that at least 98% of cells have <u>at least one</u> PE-III can be determined with the Poisson equation, by calculating the probability of zero PE-III molecules. By trial and error, we find that 4 molecules per cell, average, gives a Poisson probability of having zero proteins in a cell at about 2%.

$$p = \frac{\mu^x}{x!} e^{-\mu} = \frac{4^0}{0!} e^{-4} = 0.018$$

A value of about 4 PE-III molecules per cell, average, will thus give the observed result of having ≥98% of cells with at least 1 PE-III molecule in the cytosol.

**Number of endosomes that must release their contents in the presence of 10 µM C16-pHD108**

An average of about 4 PE-III molecules per cell is required to explain our observations. We assume here, for simplicity, that all endosomes are either 500 nm or 200 nm in diameter and contain 1.6 molecules of PE-III, or 0.1 molecules of PE-III, respectively, on average. **Therefore between 3 and 40 such endosomes would need to release their contents to achieve an average of 4 PE-III molecules per cell, consistent with our observations.** In reality, endosome sizes vary widely across this range, and includes many very small structures and a few very large structures.

1. Murk JL, Humbel BM, Ziese U, Griffith JM, Posthuma G, Slot JW, Koster AJ, Verkleij AJ, Geuze HJ, Kleijmeer MJ. Endosomal compartmentalization in three dimensions: implications for membrane fusion. Proc Natl Acad Sci U S A. 2003;100(23):13332-7.

2. Deng Q, Barbieri JT. Molecular mechanisms of the cytotoxicity of ADP-ribosylating toxins. Annu Rev Microbiol. 2008;62:271-88.
